# Long-Term Health-Related Quality of Life in German Patients with Juvenile Idiopathic Arthritis in Comparison to German General Population

**DOI:** 10.1371/journal.pone.0153267

**Published:** 2016-04-26

**Authors:** Swaantje Barth, Johannes-Peter Haas, Jenny Schlichtiger, Johannes Molz, Betty Bisdorff, Hartmut Michels, Boris Hügle, Katja Radon

**Affiliations:** 1 Institute and Outpatient Clinic for Occupational, Social and Environmental Medicine, University Hospital of Munich (LMU), Munich, Germany; 2 German Centre for Rheumatology in Children and Adolescents, Garmisch-Partenkirchen, Germany; VU University Medical Center, NETHERLANDS

## Abstract

**Objective:**

Aims of the study were to investigate health-related quality of life (HRQOL) in adult patients with former diagnosis of Juvenile Idiopathic Arthritis (JIA), to compare their HRQOL with the general population and to identify factors related to a poor outcome.

**Methods:**

In 2012, a cross-sectional survey was performed by mailing a questionnaire to a large cohort of former and current patients of the German Centre for Rheumatology in Children and Adolescents. Only adult patients (≥18 years) with a diagnosis compatible with JIA were included (n = 2592; response 66%). The questionnaire included information about HRQOL (EQ5D), disease-related questions and socio-demographics. Prevalence and 95% confidence intervals (CI) of problems with mobility, self-care, usual activities, pain and anxiety/depression were standardized to the German general population. Factors associated with low HRQOL in JIA patients were identified using logistic regression models.

**Results:**

Sixty-two percent of the study population was female; age range was 18–73 years. In all dimensions, JIA patients reported statistically significantly more problems than the general population with largest differences in the pain dimension (JIA patients 56%; 95%CI 55–58%; general population 28%; 26–29%) and the anxiety/depression dimension (28%; 27–29% vs. 4%; 4–5%). Lower HRQOL in JIA patients was associated with female sex, older age, lower level of education, still being under rheumatic treatment and disability.

**Conclusions:**

HRQOL in adult JIA patients is considerably lower than in the general population. As this cohort includes historic patients the new therapeutic schemes available today are expected to improve HRQOL in future.

## Introduction

Juvenile Idiopathic Arthritis (JIA) is the most common chronic, inflammatory, rheumatic disease in childhood and adolescence [1, 2]. It is defined as a form of arthritis with unknown etiology that begins before the age of 16 years and persists at least six weeks [3]. First symptoms may occur as early as 3–6 years of age [4]. The international annual incidence of JIA varies between 0.8 and 22.6 per 100.000 children. The global prevalence is reported between 7 and 401 per 100.000 children [5]. In Germany about 7 of 100.000 children are newly diagnosed each year [6].

Patients with active JIA often live with chronic or recurrent pain and disability [7, 8]. Outcome in adults is variable but a considerably high number of children have ongoing disease in adulthood associated with limitation in everyday life [7–13]. As so far there is no cure of JIA, treatment primarily aims to improve health-related quality of life (HRQOL) by decreasing inflammation, preserving joint function and reducing pain and recurrence [14–17]. HRQOL is a useful marker for evaluating the effectiveness of treatment and moreover is helpful in informing patients and their families regarding the prognosis of their disease. Quality of life (QOL) is defined as individual perception of life in the context of culture and value system and in relation to the individual goals, expectations, standards and concerns [18]. HRQOL concerns the physical, emotional and social aspects of QOL which are influenced by a present disease and its treatment [18]. Several studies found an impaired HRQOL in children and adolescents with JIA [19–23] as well as in JIA-affected adults [13, 24–26] however, most of these studies investigated only small cohorts of patients and did not consider long-term HRQOL [23].

The aim of this study was to investigate HRQOL in adult JIA patients with a wide age range. We compared HRQOL in JIA patients with data from German general population and identified factors associated with poor HRQOL.

## Methods

### Study design and study population

A single-centre hospital-based cross-sectional study was performed, including current and former rheumatic patients that had been admitted to the German Centre for Rheumatology in Children and Adolescents (DZKJR) between 1952 and 2010 (n = 10,580). The DZKJR is treating children with inflammatory rheumatic diseases since 1952 and is Europe`s largest specialized department for pediatrics and adolescent rheumatology. The DZKJR is established for their holistic therapy concept that comprises medical care, nursing, physiotherapy and occupational therapy as well as social-psychological aspects. A self-administered standardized questionnaire was sent in January 2012. When letters were undeliverable, addresses were researched at local registration offices (n = 5970); registration is mandatory in Germany. Written informed consent was given by participants or in case of children by their parents. Information of patient records was anonymized and de-identified prior to analysis. The ethics committee of the University Hospital of Munich (LMU) approved the study in September 2011.

### Questionnaire

The 23-item self-administered questionnaire assessed

- socio-demographic characteristics (age, sex, level of education, and current occupation) [27, 28],
- HRQOL by means of the EQ5D-3L questionnaire (German language version 1.0) and the EQ visual analogue scale (EQ-VAS) [29–31]. The EQ5D-3L questionnaire evaluates the HRQOL in the five dimensions mobility, self-care, usual activities, pain/discomfort and anxiety/depression on a three-point Likert scale ranging from no problems, moderate problems to severe problems. The EQ-VAS measures current overall state of health using a 15 cm long scale labelled from 0 to 100. Participants are asked to mark their current state of health on the scale with zero being the worst and 100 being the best possible state of health.
- Details on rheumatic disease (treatment, drugs, disability, psoriasis) to evaluate long-term outcome of the disease.

The latter questions had to be newly designed for the study and were face-validated by two experts. In addition, a pilot test was performed with six patients of the Institute and Outpatient Clinic for Occupational, Social and Environmental Medicine of the University Hospital of Munich (LMU). Thereafter, we tested logistics and acceptance of the questionnaire by sending out 100 questionnaires to a random sample of the target population.

### Data extraction from the patient files

**Fig 1:**
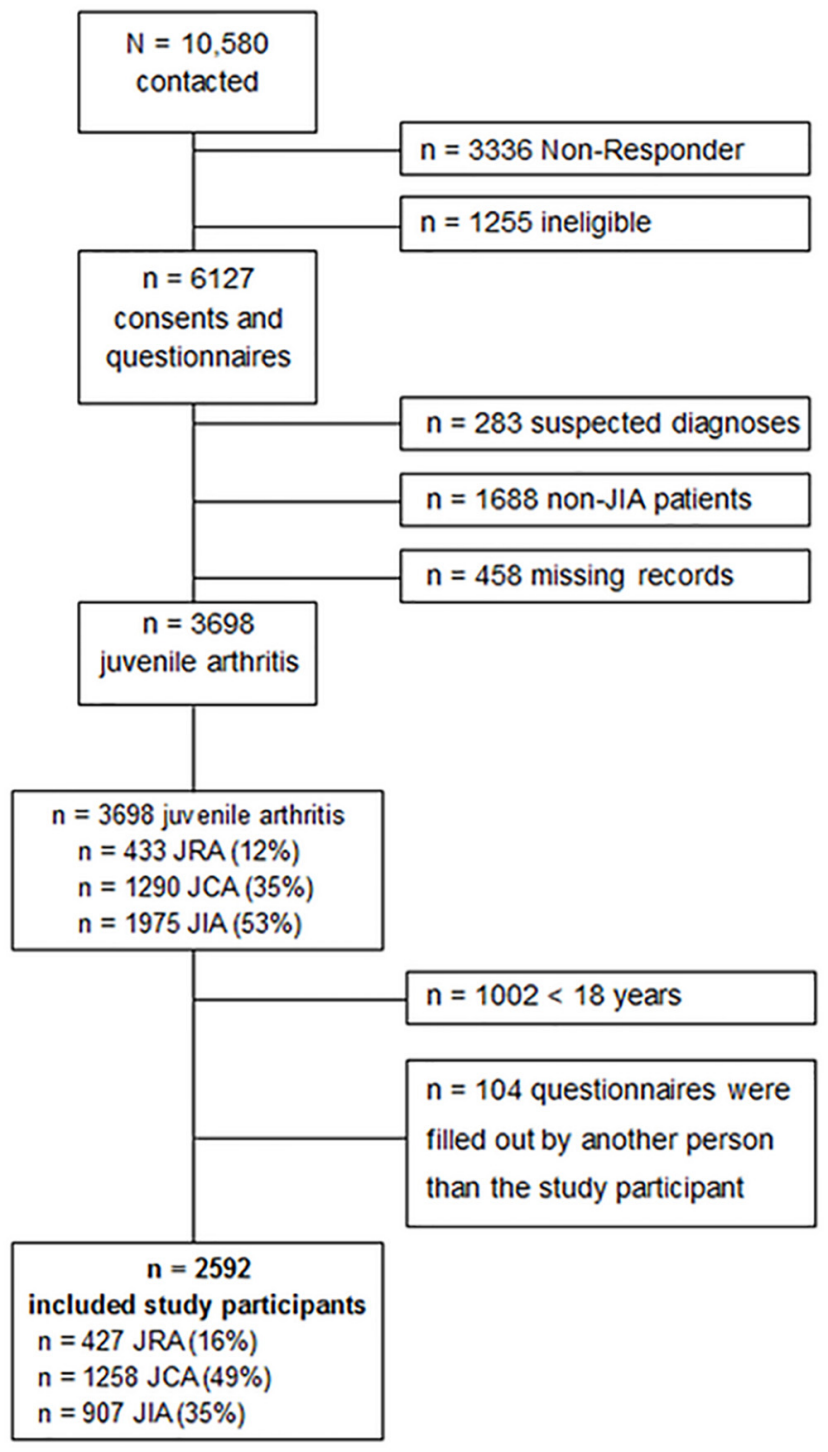
Inclusion of study participants.

In addition to the questionnaire data, date of admission to DZKJR and date of first symptoms were extracted from the medical records for all participants (Fig 1).

Furthermore, based on the patient files we identified JIA patients and excluded those suffering from other (rheumatic) diseases. As the definition of childhood chronic arthritis was changed several times since 1952 [3, 32, 33] we had to re-diagnose patients treated before 1997, the year when the current definition of JIA was put into place. This procedure has formerly been performed in order to evaluate the ILAR criteria for JIA [34]. In our definition, we intended to be as specific as possible, including only the 3698 patients with one of the following diagnoses in their record:

- diagnosis of Juvenile Rheumatoid Arthritis (JRA), Juvenile Chronic Arthritis (JCA) or Juvenile Idiopathic Arthritis (JIA)
- diagnosis of Spondylarthritis or Psoriatic Arthritis, (as these diagnoses are included in the definition of JIA but were not included in JRA and JCA definition)
- diagnosis of Rheumatoid Arthritis (RA) or Still`s disease with onset before the age of 16 years, disease duration of at least six weeks and no known cause of the symptoms.

### Variable definition

Data were double entered by two independent persons using SurveyMonkey (SurveyMonkey Inc., USA); verification was done with Synchronizer 10.0 (2000–2014, XL Consulting GmbH, Switzerland).

As socio-demographic measures we used age (years), sex (male/female) and level of education. We summarized education into high (higher school certificate, university, or college degree), medium (secondary school leaving certificate, or comparable degree) and low level of education (lower secondary education level or no degree). For pupils (n = 5) and in case of missing values (n = 48) parents highest education was used as a proxy. In case the parental level of education differed between mother and father, we used the higher level.

For details on the rheumatic disease, we asked for current treatment (yes/no), taking drugs (yes/no), having a disability card (yes/no) and if so what level of disability was registered. As common practice in Germany level of disability was measured on a scale between 0 and 100, where ≥50 indicated severe disability.

For each of the five dimensions of the EQ5D-3L questionnaire, problems were defined as either being present (moderate or severe problems) or absent (no problems). Additionally, for multiple analyses the responses to the five dimensions were summarized into the EQ5D_Index_ using the scoring algorithms (VAS-value set) of the German validation study [35]. The resulting index ranges from -1 (worst possible health status) to +1 (best possible health status). We used the first decile of the study population as cut-off to define low HRQOL. Likewise, we analyzed the responses to the EQ-VAS as continuous outcome as well as the a-priori at the first decile of the study population dichotomized EQ-VAS.

### Statistical analyses

Analyses were restricted to JIA patients being 18 years and older, as the EQ5D-3L questionnaire was only validated for adults (≥18 years) and to questionnaires which were completed by the participants themselves (Fig 1).

Socio-demographic and disease-related data as well as HRQOL were described using absolute and relative frequencies. For continuous data measures of central tendency (median values) and measures of dispersion (1.-3. quartile and range) were calculated.

In bivariate analyses, HRQOL was stratified by age, according to categories suggested by König et al. [18–24; 25–34; 35–44; 45–54; 55–64; 65–74; 75+ years) and sex and was compared to EQ5D reference data from the German general population. König et al. investigated HRQOL in 3552 adult Germans (1660 men and 1892 women). [36]. In addition, age and sex standardized prevalence with 95% confidence intervals of all HRQOL dimensions were calculated using the German general population [36] as reference.

Subsequently, we developed logistic regression analyses to identify factors associated with poor HRQOL within the population of JIA patients. For these analyses, the dichotomized EQ5D_Index_ as well as the dichotomized EQ-VAS were used as outcome variables. All socio-demographic and disease-related variables associated with the respective outcome (p_Chi²_<0.10) were simultaneously entered into the models. To rule out multi-collinearity the Variance Inflation Factor (VIF) was calculated and multi-collinearity was assumed when VIF>2 [37].

In sensitivity analyses, the five dimensions of EQ5D were used as outcome variables. Additionally, the multiple logistic regression models were repeated including one by one variables previously excluded from the final model due to multi-collinearity. Furthermore, stratified analyses for the diagnoses (JRA/JCA/JIA) were performed to investigate potential differences between these patient groups. As there are two different German value sets to calculate the EQ5D_Index_ and as recommended by the EuroQol Office for sensitivity analyses we used the German TTO-value set [38] instead of the VAS-value set [35] to generate the EQ5D_Index_.

Stata software version 12.1 (StataCorp LP, USA) was used to perform the described statistical analyses.

## Results

Questionnaires and written informed consent were returned from 6127 patients (response 66%). Of these, 3698 (60%) were JIA patients and thus eligible. Of them, 2592 patients (70%) were adults, had completed the questionnaire themselves and could therefore be included in the analyses (Fig 1). Of all included study participants 16% initially had a diagnosis of JRA (n = 427), 49% a JCA (n = 1258) and 35% had a diagnosis of JIA (n = 907) (Fig 1). For 82% of the patients the subgroup of their disease was available. The most frequent subgroup was oligoarthritis (55%), followed by polyarthritis (30%) and the systemic form (6%). All other subgroups each represented less than 4%.

### Descriptive data

Age of the patients ranged from 18 to 73 years with more than half of the patients being younger than 35 years (60%). Sixty-two percent were female and 50% had a high level of education. First symptoms occurred at a median age of 8 years (1.-3. quartile: 4–11; range: 0–15) and first admission to DZKJR was at a median age of 12 years (1.-3. quartile: 8–15; range: 1–44). Average disease duration from first symptoms until the time of the survey was 27 years (1.-3. quartile: 15–40; range: 3–70). More than half of the patients (52%) were currently receiving medical treatment because of ongoing rheumatic disease; 50% were taking medication for JIA treatment. One third of participants reported to have a disability; of these, 30% indicated a severe disability (Table 1).

**Table 1:** Socio-demographic characteristics and disease specific data of the study population.

	n_missing_	n (%)
**Total**		2592 (100)
Sex	0	
Female		1617 (62.4)
Age (years)	0	
18–24		749 (28.9)
25–34		834 (32.2)
35–44		555 (21.4)
45–54		345 (13.3)
55–76		109 (4.2)
Level of education	14	
Low		438 (17.0)
Medium		846 (32.8)
High		1294 (50.2)
Disease duration since first symptoms	1521	
Median (1./3. quartile) (range) (years)		27 (15/40) (3–70)
Age at first symptoms	1521	
Median (1./3. quartile) (range) (years)		8 (4/11) (0–15)
Currently in treatment for rheumatic disease	4	1352 (52.2)
Currently taking drugs	5	1298 (50.2)
Disability card holder	6	934 (36.1)
Severe disability	16	783 (30.4)
Psoriasis	83	249 (9.9)

### Health-related quality of life in JIA patients compared to the German general population

**Fig 2:**
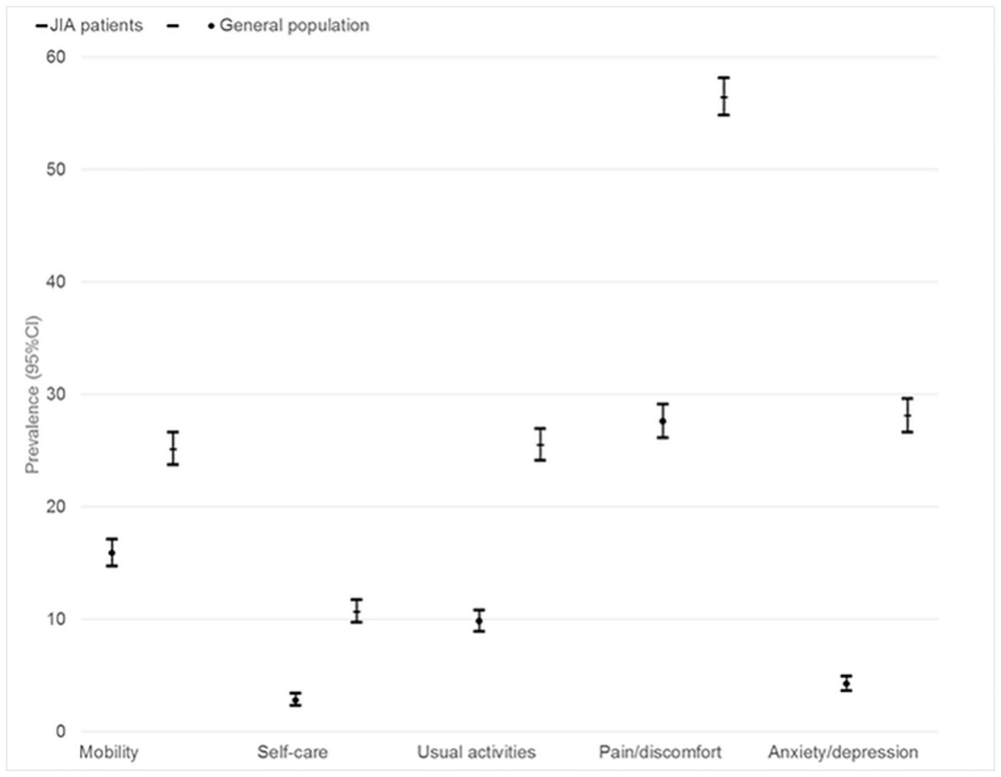
Health-related quality of life in JIA patients and the general German population. Age and gender standardized prevalence of problems in the five EQ5D dimensions with 95% confidence intervals.

Regarding HRQOL, overall age and sex standardized prevalence of problems in JIA patients was highest in the pain dimension (56%), followed by anxiety / depression (28%), limitations in usual activities (26%) and limited mobility (25%). All EQ5D dimensions were statistically significantly worse in JIA patients than in the general German population (Tables 2 and 3 and Fig 2).

**Table 2:** Age and gender stratified comparison of the EQ5D domains between JIA patients and the German general population^a^.

Men (% reporting moderate or severe problems)								
Age in years	18–24	25–34	35–44	45–54	55–64	65–74	75+	Men Overall
German general population	127	259	389	290	292	205	98	1660
JIA patients	225	296	240	170	31	13	0	975
**Mobility**								
German general population	0.8	3.1	6.4	17.2	20.9	30.2	41.8	14.9
JIA patients^b^	7.6	14.5	13.8	21.2	29.0	30.8	0	18.7
**Δ**	***6*.*8***	***11*.*4***	***7*.*3***	***3*.*9***	***8*.*1***	***0*.*5***	-	***3*.*7***
**Self-care**								
German general population	0.0	0.4	0.8	1.7	2.4	4.4	11.2	2.2
JIA patients^b^	1.3	3.0	3.3	4.7	9.7	23.1	0	6.7
**Δ**	***1*.*3***	***2*.*7***	***2*.*6***	***3*.*0***	***7*.*3***	***18*.*7***	-	***4*.*6***
**Usual activities**								
German general population	3.2	1.9	3.9	10.7	11.0	17.6	25.5	8.9
JIA patients^b^	7.1	13.5	15.0	21.2	22.6	30.8	0	17.6
**Δ**	***4*.*0***	***11*.*6***	***11*.*1***	***10*.*5***	***11*.*6***	***13*.*2***	-	**8.7**
**Pain/discomfort**								
German general population	9.5	17.8	16.5	28.3	31.9	371	41.8	24.9
JIA patients^b^	44.0	45.6	44.2	54.7	58.1	69.2	0	49.2
**Δ**	***34*.*6***	***27*.*9***	***27*.*7***	***26*.*4***	***26*.*2***	***32*.*2***	-	***24*.*2***
**Anxiety/depression**								
German general population	3.9	3.1	2.3	4.5	4.1	3.9	5.1	3.6
JIA patients^b^	15.6	17.9	17.9	27.7	19.4	15.4	0	18.3
**Δ**	***11*.*6***	***14*.*8***	***15*.*6***	***23*.*2***	***15*.*3***	***11*.*5***	-	***14*.*7***
Women (% reporting moderate or severe problems)								
Age in years	18–24	25–34	35–44	45–54	55–64	65–74	75+	Women Overall
German general population	137	292	449	347	305	213	149	1892
JIA patients	524	538	315	175	55	10	0	1617
**Mobility**								
German general population	4.4	4.8	4.2	11.5	22.3	37.6	61.1	16.8
JIA patients^b^	15.5	22.1	27.0	39.4	43.6	50.0	0	30.8
**Δ**	***11*.*1***	***17*.*3***	***22*.*8***	***27*.*9***	***21*.*3***	***12*.*4***	-	***14*.*0***
**Self-care**								
German general population	0.7	0.3	1.3	2.0	2.0	7.0	18.8	3.4
JIA patients^b^	3.2	6.3	11.1	18.9	14.6	40.0	0	14.2
**Δ**	***2*.*5***	***6*.*0***	***9*.*8***	***16*.*8***	***12*.*6***	***33*.*0***	-	***10*.*8***
**Usual activities**								
German general population	4.4	4.5	3.8	5.5	15.4	19.7	38.9	10.7
JIA patients^b^	16.6	22.1	31.1	37.7	41.8	60.0	0	32.4
**Δ**	***12*.*2***	***17*.*7***	***27*.*3***	***32*.*2***	**26.4**	***40*.*3***	-	***21*.*7***
**Pain/discomfort**								
German general population	16.1	17.8	20.9	27.1	37.7	47.4	59.7	30.0
JIA patients^b^	57.3	67.1	65.1	66.3	72.7	80.0	0	62.8
**Δ**	***41*.*2***	***49*.*3***	***44*.*1***	***39*.*2***	***35*.*0***	***32*.*6***	-	***32*.*9***
**Anxiety/depression**								
German general population	5.1	4.1	4.2	5.2	4.6	5.6	6.7	4.9
JIA patients^b^	25.8	29.7	31.4	38.3	41.8	80.0	0	36.7
**Δ**	***20*.*7***	***25*.*6***	***27*.*2***	***33*.*1***	***37*.*2***	***74*.*4***	-	***31*.*8***

Δ: Difference between German general population and JIA patients.

^a^ German data of König et al 2009;

^b^ Data for the JIA patients directly age and sex standardized to the general population.

**Table 3:** Overall comparison of problems in EQ5D domains between JIA patients and the German general population^a^.

	Overall
	% (95%-CI)
**Total (n)**	
**German general population**	3552
**JIA patients**	2592
**Mobility**	
**German general population**	15.9 (14.8;17.2)
**JIA patients**^b^	25.2 (23.7;26.6)
**Δ**	9.2
**Self-care**	
**German general population**	2.8 (2.3;3.4)
**JIA patients**^b^	10.7 (9.7;11.8)
**Δ**	7.9
**Usual activities**	
**German general population**	9.9 (8.9;10.9)
**JIA patients**^b^	25.5 (24.1;27.0)
**Δ**	15.7
**Pain/discomfort**	
**German general population**	27.6 (26.2;29.1)
**JIA patients**^b^	56.4 (54.8;58.1)
**Δ**	28.8
**Anxiety/depression**	
**German general population**	4.3 (3.6;5.0)
**JIA patients**^b^	28.1 (26.6;29.6)
**Δ**	23.8

95%-CI: 95% Confidence Interval. Δ: Difference between German general population and JIA patients.

^a^ German data of König et al 2009;

^b^ Data for the JIA patients directly age and sex standardized to the general population.

Differences between JIA patients and the general population were found to be more pronounced in women than in men (Table 2). With respect to age, problems in the pain dimension were constantly increased in JIA patients independent of age. Although at a lower level, differences in problems in mobility remained stable with increasing age. In contrast, the differences regarding the ability to carry out usual activities increased for men and women with increasing age. Differences in problems with self-care and anxiety/depression were higher in older than in younger women while they remained stable over the different age groups in men.

### Factors associated with lower health-related quality of life in JIA patients

When comparing HRQOL of JRA, JCA and JIA patients, no statistically significant differences were found (data not shown). Stratification by status of disease (active/inactive disease) revealed more problems in patients with active diseases in all dimensions of the EQ5D (p<0.001) (S2 Table).

The median EQ5DIndex of the study population was 0.902 (1.-3. quartile: 0.737–1; range 0.036–1), the EQ-VAS value 80 (1.-3. quartile: 70–90; range 0 to 100). The first decile, a priori used as cut-off to define lower HRQOL, was 0.622 for the EQ5DIndex and 50 for the EQ-VAS values.

As in the analyses of the single items of the EQ5D, women and older patients more often reported a lower HRQOL than men and younger patients (p<0.001). This was true for both the EQ5DIndex and the EQ-VAS. Likewise, a lower level of education was also related with reporting a lower HRQOL (p<0.001). Regarding disease-related factors, we found an association between treatment, taking drugs, having a disability card–especially suffering severe disability–as well as longer disease duration and both measures of lower HRQOL (p<0.001). Age at first symptoms was weakly (EQ5DIndex, p = 0.04), respectively not (EQ-VAS, p = 0.34) associated with HRQOL. Finally, patients reporting psoriasis were more likely to report a lower HRQOL (statistically significant only for EQ5DIndex, p = 0.002) (Table 4 and S1 Table). After mutual adjustment, associations were confirmed; however, Odds Ratios (OR) slightly decreased (Table 4). Associations for particular EQ5D dimensions were similar to those for the overall EQ5DIndex (S1 Table). Including variables previously excluded due to multi-collinearity in the multivariate models, the results did not change considerably (data not shown). Applying the German TTO-value set instead of the German VAS-value set to form the EQ5DIndex, only resulted in a slightly change of the median (0.887 vs. 0.901) (data not shown).

**Table 4:** Associations between general and disease specific factors and HRQOL in JIA patients. Results of bivariate analyses and logistic regression models with EQ5D_Index_ as Outcome.

	Low HRQOL (low EQ5D_Index_)			
	OR (95% CI)			
Complete cases n = 2456	% (n)	p_Chi2_	Crude OR	Adjusted OR^a^
Sex				
Male	7.63 (70)	p<0.001	1.00 (Ref.)	1.00 (Ref.)
Female	14.55 (224)		**2.06 (1.55;2.73)**	**1.74 (1.27;2.38)**
Age				
18–24 years	7.31 (52)	p<0.001	1.00 (Ref.)	1.00 (Ref.)
25–34 years	11.82 (94)		**1.70 (1.19;2.42)**	**1.63 (1.12;2.37)**
35–44 years	11.13 (58)		**1.59 (1.07;2.35)**	1.43 (0.94;2.18)
45–54 years	20.18 (66)		**3.20 (2.17;4.74)**	**3.38 (2.18;5.25)**
55–76 years	23.53 (24)		**3.90 (2.28;6.68)**	**3.72 (1.99;6.93)**
Level of education				
High	8.02 (100)	p<0.001	1.00 (Ref.)	1.00 (Ref.)
Medium	14.11 (113)		**1.88 (1.42;2.51)**	**1.70 (1.25;2.32)**
Low	19.85 (81)		**2.84 (2.07;3.90)**	**2.42 (1.70;3.45)**
Currently in treatment for rheumatism				
No	3.66 (43)	p<0.001	1.00 (Ref.)	1.00 (Ref.)
Yes	19.58 (251)		**6.40 (4.58;8.95)**	**1.88 (1.14;3.08)**
Currently taking drugs				
No	3.60 (44)	p<0.001	1.00 (Ref.)	1.00 (Ref.)
Yes	20.28 (250)		**6.81 (4.89;9.49)**	**2.98 (1.85;4.79)**
Disability card holder				
No	4.80 (75)	p<0.001	1.00 (Ref.)	1.00 (Ref.)
Yes	24.55 (219)		**6.46 (4.89;8.53)**	**2.79 (2.03;3.82)**
Severe disability^b^				
No (DoB <50)	8.09 (11)	p<0.001	n. r.	n. r.
Yes (DoB ≥50)	27.61 (206)			
No disability	4.80 (75)			
Psoriasis				
No	11.31 (250)	p = 0.002	1.00 (Ref.)	1.00 (Ref.)
Yes	17.96 (44)		**1.72 (1.21;2.44)**	1.38 (0.93;2.05)
Disease duration since first symptoms^b^				
0–5 years	18.75 (3)	p<0.001	n. r.	n. r.
6–10 years	7.14 (6)			
11–20 years	8.70 (26)			
21–40 years	12.21 (48)			
41-max years	24.20 (53)			
Age at first symptoms^b^				
0–2 years	17.50 (28)	p = 0.041	n. r.	n. r.
3–5 years	15.46 (32)			
6–8 years	13.86 (28)			
9–10 years	7.25 (10)			
11–12 years	9.26 (15)			
13–15 years	16.20 (23)			

OR: Odds Ratio, 95% CI: 95% confidence interval, Ref: reference, HRQOL: health-related quality of life, n.r.: not reported. Complete case analyses n = 2456. Low EQ VAS was defined by 1st decile of EQ VAS values (<50). Low EQ5D_Index_ was defined by 1st decile of EQ5D_Index_ values (≤0.622). The five dimensions of EQ5D were dichotomized to presence (moderate and severe problems) and absence of problems, having problems was used as outcome.

^a^ mutual adjustment for all other variables listed in the table.

^b^Variables excluded from multivariate analyses due to multi-collinearity.

## Discussion

Our study population of JIA patients retraced in adulthood reported more problems in all five dimensions of the EQ5D than the general population. This was especially true for the pain domain. In total, half of all patients were still in treatment and taking drugs because of their disease. One third of participants reported having a disability card, 30% of them indicated a severe disability. Socio-demographics (female, older age, lower education) and disease-related factors (being in treatment, taking drugs, having a disability) were main predictors of lower HRQOL.

In order to assess HRQOL in the general population we compared our results to reference data from German general population [36]. After direct standardization using the general population as reference, JIA patients still showed more problems in all EQ5D dimensions. Therefore, we concluded that JIA patients are impaired with regard to HRQOL. Nevertheless, differences in study design especially in data assessment have to be considered; we did a paper survey and König et al. [36] conducted computer-assisted personal interviews, this might result in some social desirability bias in the study by König et al. and thus under-reporting of symptoms. However, it is unlikely to explain the huge differences found between the two populations.

Some former studies which compared HRQOL of JIA patients with general population or healthy controls among children and adolescents [20, 22, 23] found an impairment of patients with JIA. In adults, there are far fewer studies that investigated HRQOL in comparison with healthy controls or general population [7, 24, 25]; their results are contradictory; moreover, only small cohorts were investigated. The variation in the results might be due to different study designs. It should be considered that most of the HRQOL studies in JIA patients used different quality of life measurement instruments and investigated different determinants; therefore a comparison of HRQOL between these studies is difficult. The cohorts differ regarding age of participants and therefore disease duration as an important predictor for HRQOL [21]. Moreover differences in the national health systems concerning treatment strategies might be relevant [39] as well as changes in treatment within the last decade.

Only one previous study by Marra et al. [40] used the EQ5D to investigate HRQOL in adult RA patients but did not compare data to general population. Overall they found, compared to our data, even lower values for HRQOL (EQ5D_Index_ mean values: 0.66 in [40] vs. 0.84 in our population). It has to be taken into account that they included only patients under current treatment who were considerably older than our patients (mean age: 62 in [40] vs. 33 years in our population). When we restricted our analyses to those patients who were currently under treatment, we obtained a mean EQ5D_Index_ of 0.77 which is rather similar to the value obtained by Marra et al. [40].

Previous studies that investigated determinants for HRQOL in adults also found that female gender [24], being older [41] and disability [25, 26] were associated with a lower HRQOL. In our cohort patients being under medical treatment and currently taking medications for JIA disease demonstrated a significant impairment of their HRQOL. Using these variables as a proxy for having an active disease, our results are in line with previous studies [10, 24]. Moreover, pain has been shown to be primarily responsible for poor HRQOL in adults [42]; for children, high levels of depression symptoms were found to be a main predictor [43]. Both are consistent with our result as patients mainly reported problems in these domains.

We found no statistically significant differences when comparing HRQOL of JRA, JCA and JIA patients. The different nomenclature (JRA/JCA/JIA) depends on when diagnosed. Therefore, one could have expected that younger patients which were more recently treated with more effective medications had a better HRQOL. However, with our data we could not confirm this hypothesis. JIA patients had an impaired HRQOL independent of age and of diagnosis (JRA/JCA/JIA).

Due to multi-collinearity we had to exclude disease duration, age at first symptoms and severe disability from the multiple logistic regression models. Although duration of disease might be a considerable determinant of HRQOL we used age as a proxy mainly because of the high number of missing values in the other two variables (each n = 1521). The many missing values occurred since date of onset of disease is difficult to define.

The EQ5D was used to describe HRQOL. The EQ5D is a standardized, non-disease-specific and easy-to-use instrument with high reliability and validity [31, 44]. Since collection and interpretation of EQ5D data is completely standardized, high objectivity of the results can be assumed. As one focus of our study was to compare HRQOL of JIA patients with that of the general population, a generic instrument appeared to be an appropriate tool. Moreover the EQ5D was previously validated for the German population [38] as well as for RA [45].

Limitations of our research may include that we may have overestimated HRQOL as we excluded the potentially more severe cases that were not able to fill out the questionnaire on their own or patients with a particular severe disease might have died in the mean-time. This was unavoidable as the EQ5D version used is not validated for proxy assessment. On the other hand we may have a priori included more severe cases and consequently underestimated HRQOL as we recruited our patients in a specialized hospital and by a possibly increased response from particularly severe cases. However, distribution of subgroups with Oligo- and Polyarthritis as the most common subgroups in our study population was similar to that of the general population. In addition, our results are not valid for patients <18 years. In future studies the now available EQ5D-Y could be used [46]. A further limitation was the cross-sectional data assessment: We illustrated long-term HRQOL by including patients with a broad age range and thus disease duration. Thereby, we cannot assess time course of HRQOL in JIA patients as this can only be assessed prospectively.

The main strength is the large study population and the relatively high participation rate. Our findings provide current data on the HRQOL of JIA patients in the age range of 18–73 years. This is a valuable basis in order to perform periodically updates considering the impact of new treatment approaches in the future [12]. Moreover, we did an age and sex standardized comparison with reference data and therefore provide valid conclusions with regard to HRQOL of JIA patients in comparison to general population.

### Conclusion

Our findings suggest that HRQOL of JIA patients is considerably lower than in the general population. Additionally older age, female gender, level of education, disability and still being under rheumatic treatment were the main predictors of poor HRQOL. New therapeutic schemes available today might help to improve HRQOL in future.

## Supporting Information

S1 Table Determinants of reporting problems in EQ5D dimensions. (DOCX)

S2 Table Health-related quality of life stratified by status of JIA (active/inactive disease). (DOCX)
