# Supplementary material for: Long-Term Health-Related Quality of Life in German Patients with Juvenile Idiopathic Arthritis in Comparison to German General Population
Source: PLoS One. 2016 Apr 26;11(4):e0153267. doi: 10.1371/journal.pone.0153267 (PMC4846020; doi:10.1371/journal.pone.0153267)
Supplement: S1 Table — (DOCX) [file pone.0153267.s001.docx]

**Online Appendix table**

Table S1: Determinants of reporting problems in EQ5D dimensions

| **Variable** | **Mobility**  OR (95% CI) | **Self-care**  OR (95% CI) | **Usual activity**  OR (95% CI) | **Pain/Discomfort**  OR (95% CI) | **Anxiety/Depression**  OR (95% CI) |
| --- | --- | --- | --- | --- | --- |
| Sex  Male  Female | 1.00 (Ref.)  1.48 (1.15;1.91) | 1.00 (Ref.)  2.12 (1.36;3.31) | 1.00 (Ref.)  1.63 (1.26;2.12) | 1.00 (Ref.)  1.47 (1.20;1.81) | 1.00 (Ref.)  1.66 (1.34;2.04) |
| Age (n=2592)  18-24 years  25-34 years  35-44 years  45-54 years  55-76 years | 1.00 (Ref.)  1.54 (1.13;2.09)  1.60 (1.14;2.25)  3.25 (2.23;4.73)  3.94 (2.28;6.83) | 1.00 (Ref.)  1.77 (0.98;3.19)  2.24 (1.21;4.13)  4.93 (2.64;9.21)  6.01 (2.69;13.44) | 1.00 (Ref.)  1.41 (1.03;1.92)  1.89 (1.35;2.65)  3.20 (2.18;4.69)  4.06 (2.30;7.15) | 1.00 (Ref.)  1.45 (1.13;1.86)  1.31 (0.99;1.74)  1.94 (1.39;2.71)  3.05 (1.79;5.21) | 1.00 (Ref.)  1.09 (0.85;1.39)  1.15 (0.87;1.52)  1.80 (1.32;2.46)  2.07 (1.29;3.32) |
| Level of education  High  Medium  Low | 1.00 (Ref.)  1.35 (1.05;1.74)  2.32 (1.72;3.14) | 1.00 (Ref.)  1.66 (1.07;2.57)  3.37 (2.12;5.36) | 1.00 (Ref.)  1.49 (1.15;1.92)  2.18 (1.60;2.97) | 1.00 (Ref.)  1.49 (1.20;1.85)  1.62 (1.22;2.16) | 1.00 (Ref.)  1.29 (1.05;1.59)  1.24 (0.95;1.62) |
| Currently in treatment  No  Yes | 1.00 (Ref.)  1.81 (1.23;2.67) | 1.00 (Ref.)  2.69 (1.21;5.98) | 1.00 (Ref.)  1.88 (1.27;2.78) | 1.00 (Ref.)  2.90 (2.18;3.86) | 1.00 (Ref.)  1.42 (1.04;1.93) |
| Currently taking drugs  No  Yes | 1.00 (Ref.)  2.48 (1.72;3.58) | 1.00 (Ref.)  2.97 (1.44;6.14) | 1.00 (Ref.)  3.41 (2.34;4.97) | 1.00 (Ref.)  3.20 (2.41;4.23) | 1.00 (Ref.)  1.39 (1.03;1.87) |
| Disability card  No  Yes | 1.00 (Ref.)  3.68 (2.85;4.74) | 1.00 (Ref.)  6.80 (3.82;12.12) | 1.00 (Ref.)  3.48 (2.70;4.47) | 1.00 (Ref.)  2.59 (2.04;3.29) | 1.00 (Ref.)  1.64 (1.32;2.04) |
| Psoriasis  No  Yes | 1.00 (Ref.)  1.19 (0.84;1.69) | 1.00 (Ref.)  1.39 (0.83;2.32) | 1.00 (Ref.)  1.18 (0.83;1.68) | 1.00 (Ref.)  1.50 (1.06;2.11) | 1.00 (Ref.)  1.55 (1.16;2.08) |

OR: Odds Ratio, 95% CI: 95% confidence interval, Ref: reference.

Complete cases analyses n=2456.
