# Supplementary material for: Long-Term Health-Related Quality of Life in German Patients with Juvenile Idiopathic Arthritis in Comparison to German General Population
Source: PLoS One. 2016 Apr 26;11(4):e0153267. doi: 10.1371/journal.pone.0153267 (PMC4846020; doi:10.1371/journal.pone.0153267)
Supplement: S2 Table — (DOCX) [file pone.0153267.s002.docx]

**Online Appendix table**

Table S2: Health-related quality of life stratified by status of JIA (active/inactive disease)

|  | Acitve JIA  n (%) | Inactive JIA  n (%) | p_Chi2_ |
| --- | --- | --- | --- |
| Mobility  no problems  moderate problems  severe problems | 1014 (69.2)  447 (30.5)  5 (0.3) | 1048 (93.6)  72 (6.4)  0 (0.0) | p<0.001 |
| Self-care  no problems  moderate problems  severe problems | 1315 (89.6)  145 (9.9)  8 (0.5) | 1108 (98.9)  10 (0.9)  2 (0.2) | p<0.001 |
| Usual activities  no problems  moderate problems  severe problems | 988 (67.5)  455 (31.1)  20 (1.4) | 1059 (94.5)  60 (5.4)  2 (0.2) | p<0.001 |
| Pain/discomfort  no problems  moderate problems  severe problems | 289 (19.8)  1083 (74.1)  89 (6.1) | 800 (71.6)  312 (27.9)  5 (0.4) | p<0.001 |
| Anxiety/depression  no problems  moderate problems  severe problems | 969 (66.3)  448 (30.6)  45 (3.1) | 934 (83.5)  176 (15.7)  9 (0.8) | p<0.001 |
